# Should All Pulpless and Impacted Teeth Be Removed?

**Published:** 1921-04

**Authors:** William H. G. Logan


### SHOULD ALL PULPLESS AND IMPACTED TEETH BE REMOVED?

BY WILLIAM H. G. LOGAN, D.D.S., M.D.

This subject will be discussed under the four following subdivisions:

1. A review of the opinions of a relationship existing between focal infections associated with teeth and systemic disease.

2. Definite statement of the essayist's opinion as to what he believes to be appropriate practice in the management of these cases until additional evidence is presented.

3. Is curettement always indicated when a tooth about which there is a chronic periapical infection is extracted?

4. Statements bearing upon the surgical management of these cases.

There occurs in dental and medical literature during the last three decades the frequent appearance of articles stating that a close relationship exists between chronic oral infections and certain systemic diseases. However, it was not until the appearance of the findings of such noted investigators as Drs. Miller, Poynton, Payne, Lambert, Hunter, Billings, Rosenow, Hartzell, Larson, Grieves, Vaughan, Gilmer, Moody and Duke that the members of our profession realized the seriousness of the relation existing between chronic oral infections and such systemic diseases as arthritis, neuritis, nephritis, the cardiovascular degenerations, neurasthenia, iritis and myalgia. Personally, I accept the basic teachings of these men upon the systemic effort of chronic focal infections and oral sepsis as being unrefuted by any group of clinicians and investigators of equal authentic standing. Having declared faith in the doctrine that chronic focal infections may be the cause of many serious systemic diseases, let us now pass to a consideration of *the essayist's opinion as to what he believes to be appropriate practice in the management of these cases until additional evidence is presented.*

(*a*) In my opinion, sufficient evidence has not been secured by either the medical or dental profession to warrant a discontinuance of the filling of all root-canals in teeth having normal supporting tissues if the size of these canals favors placing a filling to the apex—providing this is done under the rigid aseptic conditions as now practiced, unless the patient in question is suffering from some serious systemic disease that might be detrimentally influenced by even a temporary infection if one should occur at the apex of the tooth.

(*b*) Until the advocates of the rather universal filling of root-canals can present additional evidence of the permanency of controlling the infection beyond a treated tooth, it is my opinion one is not warranted in advising a patient to have the root-canal filled in a tooth about which exists a chronic periapical infection that has resulted in the destruction of the pericemental fibres. The only exception I would make to the foregoing statement (*b*) is where a patient has never suffered from a disease that might be influenced by a chronic infection, and the tooth is one that experience has taught us to consider a favorable risk for root amputation.

The results obtained in the operation of root-end amputations by even our best operators have not been such as to make the conservative practitioner promise a patient that there is any certainty that he will retain such a tooth for a given number of years, or that it positively will be free from an infected area for any length of time. However, there is some apparent clinical evidence that infection is eradicated in a certain percentage of such cases; therefore I have not abandoned root amputation entirely, but my records show that I have only performed it twice in the last ten months, and in both instances the patient was a woman and the tooth involved the superior central incisor with less than one-third of the root end affected.

With the evidence presented by our investigators and clinicians it would seem that the profession is not warranted to entirely discontinue the filling of root-canals, but we surely have passed the point where the dental profession can with justification fill all teeth having sufficient supporting structures to retain them comfortably in the jaws in spite of the fact the apex is denuded and is forming a portion of the wall of an infected field.

We shall now pass to the consideration of cases presenting with a number of pulpless teeth in which root-canal fillings have been previously placed and the patients seek advice as to their retention or extraction. For the purpose of discussion we will divide these patients into two groups.

Group 1—*Patients suffering from some disease held to be caused or influenced by chronic localized infections.*

If a patient is a sufferer from myo or endocarditis, a marked hyper or hypo blood pressure, neuritis, iritis, chronic nephritis, gastic or duodenal ulcers, for example, I would advise the extraction of all pulpless teeth held to be positively involved with chronic periapical infection—but I would not condemn every pulpless tooth diagnosed by the aid of proper radiographs as containing perfect root-canal fillings and having normal pericemental and periapical tissues. However, one feels more safe in the presence of extreme systemic disturbances, which may be accounted for by focal infection associated with teeth, to demand removal of all pulpless teeth in spite of normal hemanalysis and even though the radiographic findings do not indicate the presence of localized infection.

The pulpless tooth should be extracted without hesitation, if systemic symptoms held to be due to the presence of infection continue after you are convinced that all areas from which teeth have been removed have healed normally, and a competent diagnostician has assured you that to the best of his knowledge no other localized areas exist in the system.

GROUP 2—*Patients held by competent diagnosticians as never having suffered from any disease or condition which has a relation to chronic localized infection—yet present with radiographs outlining small periapical rarefied areas about pulpless teeth.*

It is this group that causes more anxiety than the one previously discussed, for the reason we have all had patients with a number of teeth involved with periapical rarefied areas that to the best of our judgment have existed from one-third to one-half the life of that individual. These patients give a history of never having been sick with any disease except those incident to childhood. In the management of these cases it has been my habit to explain that although they have been healthy and may remain free from disease influenced by or traceable to chronic infection, retention of these infected tracts must be recognized as a menace to their health. In spite of their previous history one can not assure them there may not come a time when lowered resistance will occur and ill health of a serious nature be the sequence of the retention of these teeth; and the only safe course to pursue is to have them removed or the root ends amputated. An explanation should be made that the present belief would indicate it is impossible to fill or refill the root-canals of a tooth and permanently control the infection beyond the apex if chronic infection has resulted in the destruction of the pericemental membrane. They must also be informed that they accept the responsibility of any ill effects that may come to them from the retention of these teeth, and we should urge upon them the importance of frequent dental radiographs and physical examinations to ascertain at an early date any departure from normal that might have a relationship to these known infections. Additional aid will be found in making differential blood counts before you can even with reservation permit them to be dismissed without advising extraction. This opinion is based upon the findings in four hundred and fifty-six consecutive blood examinations for patients presenting at my office for consultation having reference to focal infection associated with teeth.

But for fear some might place too much reliance upon hemanalysis in a single case, permit me to report that in this series neither pronounced nor moderate anemia was found to be commonly associated with chronic oral infection. Pronounced anemia was found but three times. Leucopenia was more constant than leucocytosis in pyorrhea cases where the blood findings were abnormal. Leucocytosis was present in 90.3 per cent. of cases of periapical infection without discharging sinuses, but leucopenia did occur under the same conditions in three per cent. of the cases.

Let the foregoing be not misconstrued to mean that either leucocytosis or leucopenia is always present when a periapical infection without a discharging sinus is found —we had cases of chronic infection at root ends where the blood findings were normal, if such evidence is needed, for naturally periods arise when the effect of the pathogenic bacteria and toxins is so slight that no blood change is manifested. But since severe secondary infections could occur during this period, a focal infection, though producing no characteristic blood change, must always be looked upon as a menace to the health of the patient.

*Is curettement always indicated when a tooth about which there is a chronic periapical infection is extracted?*

The necessity for a thorough curettement of certain periapical infected tracts found associated with teeth is a procedure that has been commonly accepted by oral surgeons who have written upon this subject in recent years; while the more universal curettement following the extraction of teeth involved with chronic infection is one that has come prominently before the profession within the last two or three years. This probably can be accounted for as a sequence of the rather infrequent occurrence of a tooth socket healing and leaving the old infected tract, resulting in certain instances in the re-establishment of the original systemic manifestations complained of by the patient before the teeth were removed. That this may positively occur has been established by a number of careful observers, who have opened into these unhealed areas under aseptic precautions and secured the original bacterial flora; furthermore, the patient has frequently had an exaggeration of the secondary involvement in the period immediately following this second curettement.

It occurred to me a short time before I left practice for military service in 1917 that we were in need of additional data as to the frequency with which these rarefied areas do not clear following extraction without curettement—also the frequency with which the sockets heal apparently normal to the most careful inspection over root fragments that were not removed at the time of original extraction. Therefore, for a brief time in 1917 and for the past ten months it has been my rule when seeking focal infections associated with teeth to demand that all teeth and tooth areas be radiographed to ascertain if bone formation has taken the place previously occupied by the socket or any rarefied area that existed beyond, and at the same time to learn if tooth fragments or impacted teeth are retained in the jaws.

I anticipated I would find evidence of former periapical rarefied areas quite frequently, and now and then a root with normal appearing overlying soft structures, but the facts are as follows:

In reviewing the radiographs of 35 edentulous jaws, 560 tooth areas and 1,024 areas from which teeth have been extracted, making a total of 1,584, positive outline of infected tracts was only made out 11 times, while the number of roots found was 75, and imbedded teeth 73. From this small collection one should not draw definite conclusions, but I feel one is warranted in asking those who advocate curettement of every socket involved with a chronic infected area, on what evidence they base the necessity of this procedure? I am also constrained to propound to those who not only demand the removal of the granuloma or cystic-like membrane found at a root end, but the curettement of the entire bone wall beyond the soft tissues—the question that if this is necessary for the regeneration of bone to fill in the rarefied area, why is it we do not find these infected tracts remaining more frequently after extraction of teeth without curettement?

From the foregoing discussion, I do not desire to convey the idea I believe curettement is never indicated, but I do desire to positively affirm that my experience and observation to date does not lead me to believe sufficient evidence has been presented to cause any operator to demand the curettement of every socket from which a pyorrhea or pulpless tooth is extracted. For the purpose of presenting more clearly the essayist's opinion as to when curettement is held to be unnecessary as well as when indicated, I shall first try to explain by words and then supplement with stereopticon illustrations of actual cases. The underlying base from which to decide for or against curettement is found, first, in the condition of the patient's health, and, second, the extent and character of the rarefied area.

If the patient is so debilitated that you believe the reparative process is far below normal, it would be fair to assume that curettement is indicated in the moderately sized and large areas, but it is my opinion that the smaller ones, even in such a patient, will clear off the infected process and heal without curettement. It would seem one could, with justification, state a granuloma existing to a degree equal to one-third the length of the root could be classed as a border-line case or one demanding curettement, and as the involvement diminishes from one-third the length of the root, the frequency with which curettement is indicated decreases until we reach the point where it is not necessary, unless the operator believes he is dealing with a dental cyst instead of a granuloma.

Having discussed when the indications might permit of the retention of the pulpless tooth as well as when removal seems demanded, and having found imbedded teeth and root fragments more frequently than unhealed rarefied areas, the question naturally arises, What should be our action when impacted teeth or root fragments are found? With reference to tooth fragments that show infected tracts about them or that have healed in with normal appearing overlying bone and soft structure, it would seem to me one would be justified in demanding their removal, for surely they can never be made useful and are without value and should be classed as unsafe to retain.

Whether one should always remove imbedded teeth when found has ever been a debatable question with me; yet I remove imbedded teeth when thought to have relation to systemic disease or reflected pain, but many have been left when radiographs outlined normal bone in close contact with all enamel surfaces of the tooth. In the presence of severe systemic diseases where all known localized infections have been removed without the expected benefit to the patient, I have felt it unsafe to leave even such a tooth in the jaws, as we are without sufficient evidence at this time to say that such teeth have not infected tracts about them, even though the radiographs show no rarefied areas.

Before closing this phase of the subject, I wish to ask the co-operation of those especially interested to assist in ascertaining if the systemic reaction is greater or less when these teeth are extracted and curetted or extracted and not curetted. I also seek data if there is a variance in the systemic reaction when the tooth is extracted and the curettement, if performed, is delayed for a period of five to ten days.

*Statements bearing upon the surgical management of these cases.*

Although it is possible in certain instances to curette the periapical area successfully through the tooth socket, one can always be more positive of results by opening through the buccal or labial plate. When the involvement is small we are warranted in making a u-shaped incision through the soft tissues overlying the field and reflecting the soft structures, including the periosteum, to expose the bone, then with chisel or bur remove the intervening bone—and upon completion of the operation the flap may be returned and sutured into position. If the area is extreme the flap is not returned and sutured. Without regard to whether the flap is to be returned or removed, the next step after removal of the intervening bone is to remove the granuloma or cystic membrane found. When thought to be accomplished the field is dried and examined, and if smooth, white bone walls are found you may be sure you have completed your curettement. It is not necessary, in my opinion, to remove the surface of this bone cavity, as has been recommended by some. As evidence that bone cavities go on to healing without the removal of the bone surface, I call your attention to the occurrence of complete bone regeneration, not only in the small, but in the abnormally large typical and atypical cysts found particularly in the body of the mandible. This occurs promptly, even in cases complicated by infection previous to operation.

Access to impacted teeth lying deep in the canine fossa and erupting to the palatal of the lateral or central can be gained most easily, unless the tooth is in an almost horizontal position, by making a u-shaped incision on the labial and reflecting the periosteum back. With a bur or hand chisel, go through the bone at a point above the lateral incisor until the impacted tooth is reached, then after identification a little more than one-third of the root is exposed and cut diagonally. The root portion can then be lifted from its bed. The next step is to dislodge the crown portion by enlarging the opening about the root to permit the crown to escape through the opening. If difficulty is encountered in bringing the crown portion to the opening, a blunt steel instrument can be forced through the intervening bone on the palatal surface to the point of the crown, and either by hand or mallet force, the crown portion of the tooth will be carried up through the labial opening. By this plan we do away with the necessity of leaving a painful wound within the palate.

This same procedure is applicable to teeth lodged on a horizontal line just above and to the palatal of the first bicuspid, providing there is space for the operation without encroaching upon the antrum. Of course at times an impacted tooth lies too far within the palate for the approach above the bicuspids or lateral incisor, then through necessity we must enter through the palatal tissues. Without injury to the large blood vessels, a large u-shaped flap can be lifted within the palate that will permit the removal of the tooth lodged in this area and then sutured into position if conditions warrant it. In certain cases the danger of secondary hemorrhage is too great to permit suturing the flap back into position throughout its entire length. In such instances it is appropriate to make an opening through the flap immediately overlying the deepest point of the bone cavity, and through this, control your hemorrhage with a gauze pack, which is to be removed in twenty-four to forty-eight hours and replaced if necessary. If you have been able to judge the location of the impacted tooth previous to making your flap, it will be possible to insert the gauze at the line of incision if needed to control the secondary hemorrhage. Unerupted teeth, located in the mandible, should be approached from the labial or buccal by preference.

Although general outlines have been suggested for the management of patients presenting with pulpless and impacted teeth, I desire to emphasize the importance of considering every case as an individual one which demands thorough investigation before a decision can be reached as to the proper course to pursue, for the man who undertakes to cure human ills by rule of thumb will frequently go far astray.

In closing, the prophecy is here made that chronic infection associated with teeth will not be effectively managed until the dentist's education includes more of bacteriology and general pathology, and the physician's knowledge of dental pathology is more comprehensive. But until this much needed state of affairs is attained, can we not agree with William Hunter, author of the term "oral sepsis," when he affirms the treatment of the lesions of strepto and staphylococci found in the mouth, throat, nose and accessory sinuses belongs to no one department of medicine or surgery, but is common ground upon which the general practitioner of dentistry and medicine, physician and surgeon, eye, ear, nose and throat specialist, and lastly the internist and oral surgeon all meet with equal responsibility.

RADIOGRAPHIC FINDINGS IN THIRTY-FIVE EDENTULOUS JAWS

| | |
|---|---|
| Number of tooth areas examined | 560 |
| Root fragments with normal appearing overlying tissues having definite periapical rarefied areas | 5 |
| Root fragments with normal appearing overlying tissues without periapical rarefied areas | 3 |
| Rarefied areas remaining unhealed after the removal of teeth | 2 |
| Foreign bodies without rarefied areas | 0 |
| Foreign bodies with rarefied areas | 0 |
| Imbedded teeth | 2 |
| Percentage of tooth sockets that did not heal normally where the entire tooth was removed | 4-10 |

RADIOGRAPHIC FINDINGS IN JAWS CONTAINING SOME NORMAL TEETH

| | |
|---|---|
| Number of tooth areas examined | 1,024 |
| Root fragments with normal appearing overlying tissues having definite periapical rarefied areas | 47 |
| Root fragments with normal appearing overlying tissues without periapical rarefied areas | 20 |
| Rarefied areas remaining unhealed after the removal of teeth | 9 |
| Foreign bodies without rarefied areas | 9 |
| Foreign bodies with rarefied areas | 0 |
| Imbedded teeth | 71 |
| Percentage of tooth sockets that did not heal normally where the entire tooth was removed | 9-10 |

—*Journal N. D. A.*

